# Placement of a modified cannula in the innominate vein for sufficient drainage during the bidirectional Glenn shunt procedure without cardiopulmonary bypass

**DOI:** 10.1186/s13019-015-0341-7

**Published:** 2015-10-27

**Authors:** Liang-Wan Chen, Xiao-Fu Dai, Xi-Jie Wu, Qi-Min Wang

**Affiliations:** Department of Cardiac Surgery, Union Hospital, Fujian Medical University, Fuzhou, Fujian 350001 China

**Keywords:** Congenital heart disease, Bidirectional Glenn shunt, Modified cannula

## Abstract

During the bidirectional Glenn shunt procedure in small infants, the standard right-angle venous cannula is frequently placed in the innominate vein for establishing the temporary veno-atrial bypass without cardiopulmonary bypass, but it should be small enough to allow flow to pass around it from the internal jugular vein opposite to the side the cannula is directed. Small cannula may induce the inadequacy of venous drainage. We developed a modified right-angle venous cannula and placed it within the innominate vein for sufficient venous drainage. The standard right-angle venous cannula was simply modified by an oval open on the top of the external curvature. Our initial application demonstrated that this modified venous cannula provides better venous drainage during the bidirectional Glenn shunt procedure without cardiopulmonary bypass in small infants.

## Background

Bidirectional Glenn shunt is one of the palliative procedures in the single ventricle and pulmonary stenosis complex. This procedure can be performed without using cardiopulmonary bypass as Lamberti firstly reported [1]. For a wide patent cavopulmonary anastomosis, the superior vena cava (SVC) should be clamped while a venous cannula is placed to drain the SVC blood [1]. In small infants, the venous cannula may interfere with the cavopulmonary anastomosis if it is placed in the distal part of the SVC because of the limited length of the SVC. In an effort to reduce such problem, the standard right-angle venous cannula is frequently placed in the innominate vein, but it should be small enough to allow flow to pass around it from the internal jugular vein opposite to the side the cannula is directed [2]. Small cannula may induce the inadequacy of venous drainage, which could cause hemodynamic instability and the danger of cerebral edema during the procedure [3]. Recently, we developed a modified right-angle venous cannula and placed it within the innominate vein for sufficient venous drainage during the bidirectional Glenn shunt procedure in small infants.

## Case presentation

### Modification of the standard right-angle venous cannula

**Fig. 1:**
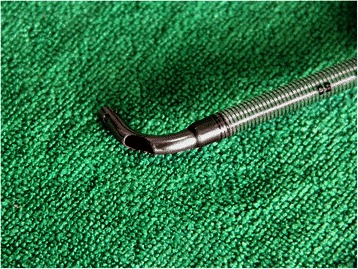
An oval open was created on the top of the external curvature in the standard right-angle venous cannula, resulting in a bidirectional right-angle venous cannula being constructed

In the standard right-angle venous cannula, an oval open was created on the top of the external curvature and it’s long axis should lie in the long axis of the cannula. The width of the oval open should be equal to the diameter of the tip of the right-angle cannula and the length should be double it’s width. After the oval open was finished, a modified right-angle venous cannula was constructed, called the bidirectional right-angle venous cannula (Fig. 1). The modified bidirectional right-angle venous cannula was locally handcrafted and sterilized.

### Surgical technique

**Fig. 2:**
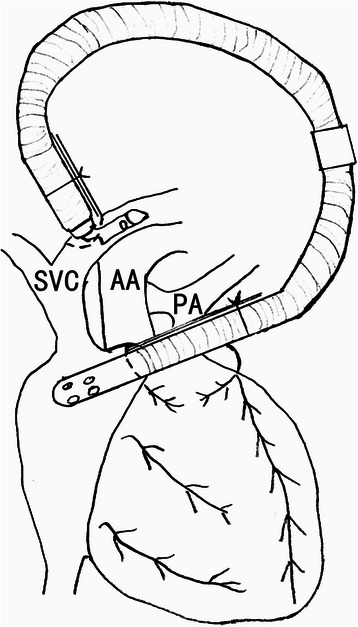
The bidirectional right-angle venous cannula was placed within the innominate vein for establishing the temporary veno-atrial bypass during the bidirectional Glenn shunt procedure in small infants. AA: ascending aorta; PA: pulmonary atery; SVC: superior vena cava; DVV: descending vertical vein

After median sternotomy and pericardial open, the SVC was dissected and isolated from atrial end to innominate vein junction, and azygos vein was ligated and divided. The right pulmonary artery was free from the bifurcation to the hilar region. The infant was heparinized (2 mg/kg). Through the purse string sutures placed on the innominate vein and the right atrial appendage, the bidirectional venous cannula and a straight venous cannula were placed within the innomonate vein and the right atrium respectively. Then, those two venous cannulae were fill up with blood to prevent air embolism, and their connection resulted in establishing the temporary veno-atrial bypass (Fig. 2). The SVC was clamped just below it’s innominate vein junction and transected just above the atrial end without damaging the sinus node. The atrial end of the SVC was closed. The right pulmonary artery was occluded at it’s proximal and distal ends with vascular clamps and opened at it’s superior aspect. As large an anastomosis as possible was then fashioned between the distal end of this SVC and the right pulmonary artery (end to side), using a running 6–0 absorbable suture. After establishing the cavopulmonary shunt, the clamps were removed and the temporary shunt was disconnected. Then, the cannulae were removed and the purse-string suture were tied. Completion of the operation was accomplished in the usual fashion.

## Discussion

From July 2011 through May 2014, 19 infants with the single ventricle and pulmonary stenosis complex and single right superior vena cava underwent the bidirectional Glenn shunt procedure at our department. In the first 10 consecutive infants (group 1), the standard right-angle venous cannula was placed in the innominate vein for establishing the temporary veno-atrial bypass. In the next 9 infants (group 2), the bidirectional right-angle cannula was used. With the use of our venous cannula, we have observed a significant reduction in the right internal jugular venous pressure from 24.50 ± 2.99 mmHg in group 1 to 21.11 ± 1.96 mmHg in group 2 (*p* < 0.05).This preliminary result suggests that our bidirectional venous cannula placed in the innominate vein may play more sufficient venous drainage than the standard right-angle venous cannula. Two major reasons might contribute to this consequence. First, when the standard right-angle cannula is placed in the innominate vein, the blood flow from the internal jugular vein opposite to the side the cannula is directed must pass around the part of the cannula inside the vein before it is drained. Second, a larger bidirectional venous cannula can be safely used while a larger standard right-angle venous cannula may cause innominate venous obstruction. In group 2, the time for completion of the cavopulmonary shunt was 25 min (range, 10 to 21 min). All the infants tolerated the procedure well without any hemodynamic compromise. On clinical examination in the immediate postoperative period, there was no facial edema. All infants underwent a routine neurological examination on the second postoperative day and did not show any abnormality. There was no hospital mortality. Postoperative echocardiogram before discharge showed functioning Glenn shunt without any obstruction in all infants. Baseline characteristics between groups were summarized in Table 1.

**Table 1:** Baseline characteristics between groups

	Group1 (*n* = 10)	Group 2 (*n* = 9)
Gender (male/female)	6/4	5/4
Ages (months)	9.1 (3–18)	8.9 (2–19)
Weight (kg)	6.5 (4.1-14.5)	6.3 (4.5-15.1)
Single ventricle and pulmonary stenosis complex and single right superior vena cava (*n*)	10	9
SVC pressure (mmhg)^*^	24.50 ± 2.99	21.11 ± 1.96
Operating time (min)	26 (11–33)	25 (10–31)
Postop facial edema or neurological abnormality (*n*)	0	0
Glenn shunt with obstruction during follow-up (*n*)	0	0

*Group 1- BDG using standard venous cannula in innominate vein, Group 2-BDG using modified venous cannula in innominate vein

*SVC pressure (mmhg) *p* < 0.05

## Conclusion

We present a simple modification of the standard right-angle venous cannula to effectively provide more sufficient venous drainage during the bidirectional Glenn shunt procedure in small infants.

## Consent

Written informed consent was obtained from the patient for publication of this Case report and any accompanying images. A copy of the written consent is available for review by the Editor-in-Chief of this journal. This case study was approved by Institutional Review Board for Union Hospital, FuJian Medical University.

## Abbreviation

svc: Superior vena cava
